# Significance of HPV-58 Infection in Women Who Are HPV-Positive, Cytology-Negative and Living in a Country with a High Prevalence of HPV-58 Infection

**DOI:** 10.1371/journal.pone.0058678

**Published:** 2013-03-07

**Authors:** Joon Seon Song, Eun Ju Kim, Jene Choi, Gyungyub Gong, Chang Ohk Sung

**Affiliations:** Department of Pathology, Asan Medical Center, University of Ulsan College of Medicine, Seoul, Korea; Albert Einstein College of Medicine, New York

## Abstract

**Purpose:**

Cervical cytology and human papillomavirus (HPV) DNA co-testing is recommended as a screening method for detecting cervical lesions. However, for women who are HPV-positive but cytology-negative, the appropriate management and significance of HPV-58 infection remain unknown.

**Methods:**

This study of prevalent HPV detected at baseline with a median follow-up of 3.2 years evaluated the risk factors associated with cervical abnormalities and assessed the significance of HPV-58 infection. A total of 265 women were enrolled. All high-grade squamous intraepithelial lesions (HSIL) that were detected by cytology were confirmed by histology. Histological diagnoses of cervical intraepithelial neoplasia 2/3 were classified as HSIL. Women were classified into four groups according to the HPV genotype that was detected at their first visit: HPV-58 (n = 27), HPV-16 (n = 52; 3 women had HPV-58 co-infection), ten other high risk (HR) types (n = 79), or low/undetermined risk types (n = 107).

**Results:**

Of 265 women, 20 (7.5%) had HSIL on their follow-up examinations. There were significant differences in the cumulative incidence of HSIL between the four groups (*p*<0.001). The 5-year cumulative incidence rates of HSIL were 34.0% (95% CI: 17.3–59.8%) in HPV-58 positive cases, 28.0% (95% CI: 13.8–51.6) in HPV-16 positive cases, 5.5% (95% CI: 2.1–14.0%) in one of the ten other types of HR-HPV positive cases, and 0% in women with low/undetermined risk HPV. When seen in women with HR-HPV (n = 158), persistent HPV infection was a significant factor associated with the development of HSIL (hazard ratio = 15.459, 95% CI: 2.042–117.045). Women with HPV-58 had a higher risk (hazard ratio = 5.260, 95% CI: 1.538–17.987) for the development of HSIL than women with HPV-16 (hazard ratio = 3.822, 95% CI: 1.176–12.424) in comparison with women with other types of HR-HPV.

**Conclusion:**

HPV-58 has a high association with the development of HSIL in women who are HPV-positive and cytology-negative.

## Introduction

The use of the Papanicolaou (Pap) smear as a screen for cervical cancer has significantly decreased the incidence of cervical cancer and its associated mortality in recent years. However, the Pap smear has a low sensitivity (<70%) for detecting high-grade squamous intraepithelial lesions (HSIL), which has raised a number of issues regarding the need for additional methods to improve detection [1], [2]. Therefore, incorporation of human papillomavirus (HPV) DNA testing is recommended in combination with the Pap test in order to improve the detection of precancerous cervical lesions [1]. The use of HPV DNA testing in conjunction with the Pap test has significantly increased the sensitivity of screening for HSIL [3]. However, the combination of HPV DNA testing and cytology has raised a new issue regarding the management of women who are HPV-positive but cytology-negative. In these cases, the current recommendations include two options: 1) repeated co-testing in 12 months; or 2) immediate HPV genotype-specific testing for HPV-16 alone or for HPV-16 and/or -18 [4]. Of these options, repeated co-testing is the preferred option for most women because most transient HPV infections are cleared within 12 months by the host immune system [4], [5], [6], [7]. In addition, the short-term risk of developing HSIL is low in this population [4], [8]. The estimated 12-month risk of developing HSIL+ in HPV-positive and cytology-negative women ranges between 0.8–4.1% [4]. However, there are limited studies that have identified the risk factors for the development of HSIL in women who are HPV-positive but cytology-negative on follow-up examinations.

HPV genotype-specific testing for HPV-16 and/or -18 (option 2) in HPV-positive and cytology-negative women may be an ineffective screening strategy for women in East Asia because there are significant differences in the distribution of HPV genotypes between Western and Eastern populations. Specifically, HPV-58, which is associated with a high risk of developing cervical dysplasia and cervical cancer, is rare worldwide but is commonly found in East Asia [9]. Overall HPV-58 is the third most common oncogenic type in Asia, but causes only 3.3% of all global cervical cancer cases [9], [10], [11]. In Korea, HPV-58 is the second most common type diagnosed in women with abnormal cytological specimens (10.8% of all abnormal cytological specimens) [12].

HPV-16 and/or -18 positivity is associated with a significant risk of developing HSIL in HPV-positive and cytology-negative women [13], [14], [15]. However, the significance of HPV-58 detection in women who are HPV-positive and cytology-negative has not been studied extensively.

In this study, we evaluated the risk factors associated with cervical abnormalities in women with HPV-positive but cytology-negative results and assessed the significance of HPV-58 infection in women living in a country with a high prevalence of HPV-58 infection.

## Materials and Methods

### Patients

This study was approved by the Institutional Review Board (IRB) of Asan Medical Center, Seoul, Korea. Given that this was a retrospective study that used registry data gathered by Asan Medical Center, the IRB waived the need for written informed consent. Data were anonymously analyzed. Our institute processed approximately 50,000 Pap tests per year, of which > 95% are SurePath liquid-based preparations. In total, 3246 women who were co-tested using cytology and HPV genotyping between December 2005 and September 2010 at Asan Medical Center were selected after searching our electronic pathology database. Of these 3246 patients who underwent co-testing, 377 (11.6%) were determined to be cytology-negative but HPV-positive. Among these 377 patients, 94 were excluded due to being lost to follow-up or having an inadequate follow-up period (i.e., these women only received short-term follow-up examinations for < 1 year without histological confirmation). However, the excluded women were not different in terms of certain characteristics, such as HR-HPV distribution, in comparison with the women enrolled in this study. Of the remaining 283 women, 18 women < 30 years of age were also excluded from the study. Finally, a total of 265 women who were HPV-positive but cytology-negative at the initial assessment with no previous cytologic abnormalities were included in the study. Women who did not undergo colposcopy, conization or hysterectomy were followed using cytology smears at 6–12 month intervals for > 1 year. All cervical smears were prepared using SurePath liquid-based preparations (TriPath Imaging, Burlington, NC, USA). Data collection was stopped when HSIL positivity was detected or when the patient underwent biopsy, conization, or hysterectomy during the follow-up period. The median follow-up period was 3.2 years (range: 0.04–6.26 years). The occurrence of low-grade squamous intraepithelial lesions (LSIL) and HSIL during the follow-up period was considered indicative of cervical abnormalities. Of a total of 265 women, all 20 women with HSIL, 30 of 62 women with LSIL, and 8 of 187 women with no cervical abnormality on follow-up cytology underwent further histological examination, such as colposcopic biopsy, conization, or hysterectomy. For this study, histological diagnoses of cervical intraepithelial neoplasia (CIN) 1 were classified as LSIL, and diagnoses of CIN 2 and 3 were classified as HSIL. Of 265 women included in this study, 193 were consistently assessed using repeated HPV testing for > 1 year as well as cytology. The remaining 72 women were assessed by cytology only during the follow-up period after the initial co-testing. Persistent HPV infection was assessed in the 193 women who had repeated HPV tests for > 1 year, and it was defined as the detection of the same HPV genotype as seen at baseline on ≥ 2 consecutive HPV tests performed for greater than 1 year. The presence of multiple infections was defined as the presence of multiple HPV types (≥ 2 genotypes) on the initial HPV test. To determine HPV-58 oncogenicity, the HPV genotypes detected at the first visit were grouped into four categories: HPV-16 positive, HPV-58 positive (either alone or in combination with other types, but excluding HPV-16), the remaining ten other HR-types (excluding any HPV-16, and -58), and low/undetermined risk types. There were three women who were diagnosed with HPV-16 and HPV-58 who were classified as HPV-16 positive in order to identify the risk of HSIL development in women infected with HPV-58. The characteristics of the women with HPV-positive and cytology-negative results are summarized in Table 1.

**Table 1:** Baseline characteristics of the 265 women with HPV-positive, cytology-negative results.

	Patients	
Characteristics	Number	%
All patients	265	100
Age (years)		
Median, years (SD)	46 (8.7)	
Range	30–71	
HPV	265	100
low/undetermined risk	107	40.4
high risk‡	158	59.6
Multiple infection	265	100
yes	79	29.8
no	186	70.2
Persistent infection#	193	72.8
yes	84	43.5
no	109	56.5
High risk genotype	158	59.6
16†	52	32.9
58	27	17.1
others	79	50.0
Incident LSIL	265	100
yes	62	23.4
no	203	76.6
Incident HSIL	265	100
yes	20	7.5
no	245	92.5

SD, standard deviation; HPV, human papillomavirus; LSIL, low grade squamous intraepithelial lesion; HSIL, high grade squamous intraepithelial lesion.

# defined as the detection of the same HPV genotype as seen at baseline on ≥ 2 consecutive HPV tests performed for greater than 1 year.

‡ include 16, 18, 31, 33, 35, 39, 45, 51, 52, 56, 58, and 59.

† include 3 women with HPV-58 co-infection.

### HPV detection

HPV testing was performed using the MyHPV DNA microchip (Mygene Co., Seoul, Korea) in accordance with the manufacturer’s protocols. Briefly, samples for HPV testing were separately collected from Pap smears using the endocervical brushes that were supplied with the MyHPV Chip kit. DNA was isolated using an isolation kit (MyGene), and nested PCR was performed for gene amplification. For the first PCR, a mixture of two sets of primers—MY09 (5′-CGT CCM ARR GGA WAC TGA TC-3′; 20 pmol) and MY11 (5′-GCM CAG GGW CAT AAY AAT GG-3′; 20 pmol)—was used. For the second PCR, the HPV primers GP5+ (5′-TTT GTT ACT GTG GTA GAT ACT AC-3′; 20 pmol) and GP6+ (5′-GAA AAA TAA ACT GTA AAT CAT ATT C-3′; 20 pmol) were used. β-Globin PCR amplification was performed as the internal control. The PCR products were subjected to electrophoresis on a 3% agarose gel. First PCR products were 450 bp and second PCR products were 150 bp. β-Globin PCR products were 250 bp. After 1^st^ PCR, 2ul of PCR products were used for 2^nd^ PCR. Ten microliters of the second PCR product was denatured for 5 minutes at 95°C and left in ice water for 5 minutes. The product was then mixed with the hybridization buffer (MyGene). This mixture was spotted on the DNA chip and subjected to a hybridization reaction for one hour in a hybridization chamber (Vision Scientific, Seoul, Korea) at 43°C. After hybridization, the product was rinsed twice with buffer I (2× standard saline citrate [SSC], 0.1% sodium dodecyl sulfate [SDS]), twice with buffer II (0.2× SSC), and once with buffer III (0.1× SSC) for 5 minutes each. Hybridized HPV DNA was identified using a model G4000 scanner (GSI Lumonics, Billerica, MA, USA). A pair of oligonucleotide probes for each of the 24 HPV types was attached to each DNA chip. When a pair of specific probes elicited a fluorescence response, the assay was said to be positive for that type of HPV. The DNA microchip used is capable of identifying specific HPV genotypes and multiple HPV infections. The HPV DNA microchip included 43 genotypes, including HPV-16, 18, 31, 33, 35, 39, 45, 51, 52, 56, 58, 59, 68, 82, 26, 53, 66, 67, 69, 70, 73, 6, 11, 34, 40, 42, 43, 44, 54, 55, 61, 62, 72, 81, 7, 10, 27, 30, 32, 57, 83, 84, and 91. All 43 subtypes of HPV are detectable on the chip. We considered HPV-16, 18, 31, 33, 35, 39, 45, 51, 52, 56, 58, and 59 as types of HR-HPV [16], and the remaining types as low/undetermined risk.

### Statistical analysis

Curves of the cumulative LSIL and HSIL incidence rates were generated using the Kaplan-Meier method, and were compared using the log-rank test. Cox regression analysis was performed to evaluate the risk factors associated with HSIL, including age, persistent infection, multiple infection, and HPV genotype. The hazard ratios and 95% confidence intervals (CI) were assessed for each factor. All tests were two-sided, and *p-*values < 0.05 were considered statistically significant. Statistical analyses were performed using Stata/IC statistical software version 12 (StataCorp Ltd., College Station, TX, USA).

## Results

### Characteristics of the study population

**Figure 1:**
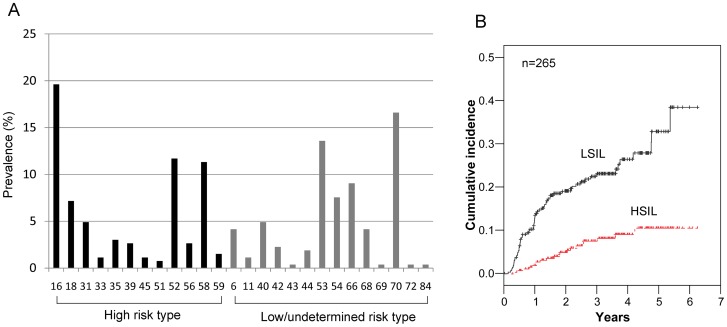
The prevalence rates of HPV genotypes and cervical abnormalities in women with HPV-positive and cytology-negative results. The prevalence rates of different HPV genotypes in 265 women with HPV-positive and cytology-negative results (A). Cumulative incidence rates of low-grade squamous intraepithelial lesions (LSIL) and high-grade squamous intraepithelial lesions (HSIL) based on 265 women with HPV-positive including high-risk and low-risk types (B).

A total of 265 women with a median age of 46 years (range: 30–71 years) who were HPV-positive but cytology-negative at baseline were included in the analysis. HR-HPV infection was detected in 158 (59.6%) women and low/undetermined risk HPV infection was detected in 107 (40.4%) women at their initial HPV tests. Of the 265 women analyzed, a single HPV type was detected in 190 (71.7%) women and ≥ 2 HPV types were detected in 79 (29.8%) women. Of these, 60 women had 2 types, 16 women had 3 types, 1 woman had 4 types, and 2 women had 5 types of HPV. HPV-16 was the most common genotype detected (19.6%; 52 of 265 women), either alone or in combination with other types, followed by HPV-70 (16.6%; 44/265) and HPV-53 (13.6%; 36/265). The prevalence of HPV-18 was 7.5% (20/265). The prevalence of HPV-58, which is known to be a high prevalence genotype in East Asia, was present in 11.3% (30/265) of the study population. The prevalence of the remaining types of HPV included HPV-52 (11.7%; 31/265), HPV-66 (9.1%; 24/265), and HPV-54 (7.5%; 20/265). The prevalence of HPV-31, 33, 35, 39, 45, 51, 56, 59, 6, 11, 40, 42, 43, 44, 68, 69, 72, and 84 were less than 5%. The prevalence of each genotype is shown in Figure 1A.

### Incidence of cervical abnormalities on follow-up examinations

During the follow-up period, LSIL was detected in 23.4% of women (62 of 265) and HSIL was detected in 7.5% of women (20 of 265). The cumulative incidence rates of LSIL and HSIL among the enrolled 265 women are shown in Figure 1B. For LSIL, the 1, 2, and 5-year cumulative incidence rates were 12.0% (95% CI: 8.6–16.7), 19.5% (95% CI: 15.0–25.0), and 31.7% (95% CI: 24.1–40.9), respectively. For HSIL, the 1, 2, and 5-year cumulative incidence rates were 1.9% (95% CI: 0.8–4.6%), 5.0% (95% CI: 2.8–8.6%), and 11.1% (95% CI: 6.8–17.8%), respectively. All HSILs were detected within 5 years of receiving follow-up examinations. When restricted to women with HR-HPV infection (n = 158), the 1, 2, and 5-year cumulative incidence rates for HSIL were 3.3% (95% CI: 1.4–7.7%), 8.4% (95% CI: 4.8–14.3%), and 18.1% (95% CI: 11.4–28.2%), respectively.

### Risk assessment of LSIL in HPV-positive and cytology-negative cases

Of the risk factors that were assessed, the development of LSIL during follow-up was associated with persistent LR-HPV infection (hazard ratio = 2.280, 95% CI: 1.003–5.184, *p* = 0.049). Persistent HR-HPV infection also tended to show an increased risk of LSIL development, but was not statistically significant (hazard ratio = 1.343, 95% CI: 0.985–1.831, *p* = 0.062). Persistent infection was present in 84 (43.5%) of 193 women. Among them, 63 had persistent HR-HPV infection and the remaining 21 had low/undetermined risk-HPV infection. The remaining risk factors, including age (≤ 45 vs. > 45 old; *p* = 0.658), HPV type (HR vs. low/undetermined; *p* = 0.997), and multiple infections (*p* = 0.759) were not significantly different in terms of the incidence of LSIL.

### Risk assessment of HSIL in HPV-positive, cytology-negative women

Of the risk factors that were assessed, HR-HPV (hazard ratio = 47.979, 95% CI: 1.209–1904.472, *p* = 0.039) and persistent HR-HPV infection (hazard ratio = 5.153, 95% CI: 1.872–14.179, *p = *0.002) were significantly associated with the development of HSIL. HSIL was also not detected in women with low/undetermined risk HPV, even in women with persistent low/undetermined risk HPV infection, and all cases of HSIL developed in women with HR-HPV infection. Older age (> 45 years old) (hazard ratio = 1.574, 95% CI: 0.627–3.947, *p* = 0.334) and multiple HPV infections (hazard ratio = 2.051, 95% CI: 0.849–4.951, *p* = 0.110) tended to be associated with a higher incidence of HSIL, but these relationships were not statistically significant.

When seen in women with HR-HPV infection, persistent HPV infection was still a significant factor associated with the development of HSIL (hazard ratio = 15.459, 95% CI: 2.042–117.045, *p* = 0.008) (Table 2). The 5-year cumulative incidence rate of HSIL was 32.3% (95% CI: 19.7–50.2%) among women with persistent HPV infection, but was only 1.8% (95% CI: 0.3–11.9%) among women with transient HPV infection. Interestingly, when using the classic classifications for HR-HPV oncogenic groups (HPV-16 and/or -18 vs. ten other HR types), the incidence of HSIL did not differ between them (hazard ratio = 1.103, 95% CI: 0.457–2.662, *p* = 0.828), which suggests that one of the other ten types of HR-HPV is a major oncogenic type of HPV. The 5-year cumulative incidence rate of HSIL was 21.2% (95% CI: 10.2–40.9%) in HPV-16 and/or -18 positive cases, but 15.8% (95% CI: 8.9–27.2%) in women with the other ten types of HR-HPV.

**Table 2:** Risk assessment of each factor for the development of HSIL in women who are high risk HPV-positive and cytology-negative.

	Univariate analysis			Multivariate analysis		
Variable	HR	95% CI	*P* value	HR	95% CI	*P* value
Age (yrs)						
≤45 vs >45	1.526	0.608-3.830	0.368	1.115	0.387–3.207	0.840
Multiple infection						
no vs yes	1.378	0.570–3.329	0.477	0.612	0.214–1.751	0.360
Persistent infection						
no vs yes	15.459	2.042–117.045	0.008	14.248	1.861–109.115	0.011
HPV type						
other 10 HR types	1	reference		1	reference	
type 16	3.822	1.176–12.424	0.026	3.536	0.906–13.796	0.069
type 58	5.260	1.538–17.987	0.008	4.244	1.013–17.780	0.048

HSIL, high grade squamous intraepithelial lesion; HPV, human papillomavirus; HR, hazard ratio; CI, confidence interval

### HPV-58 is a major risk factor for development of HSIL in HPV-positive and cytology-negative women

**Figure 2:**
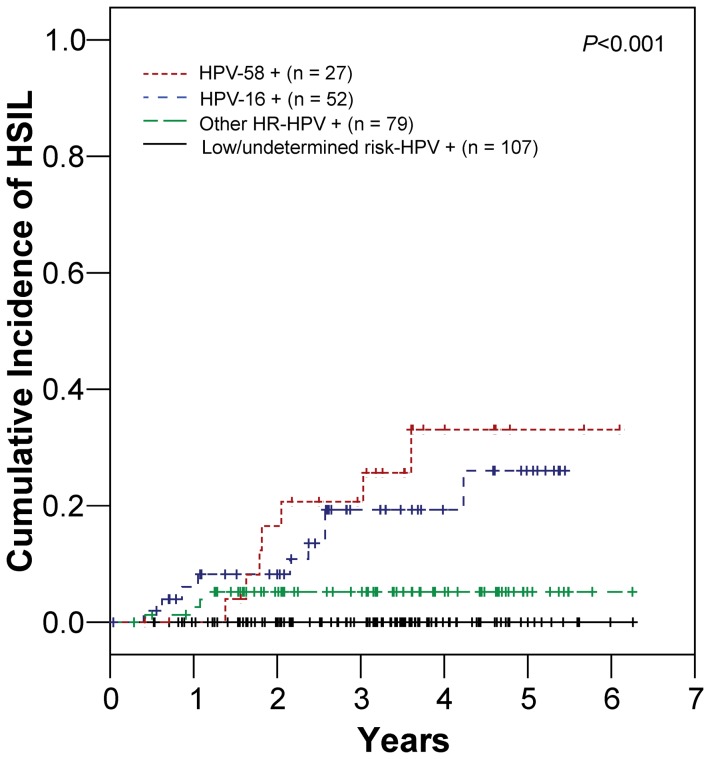
Cumulative incidence rates of high-grade squamous intraepithelial lesions (HSIL) according to the HPV genotypes. Women with HPV-positive and cytology-negative results were classified into 4 groups depending on the HPV genotype detected on the first examination. Significant differences in the incidence of HSIL were found between the 4 groups. Note that there is a higher risk of development of HSIL in women with HPV-58 infection.

When all women were classified into 4 groups depending on the HPV genotype at baseline, significant differences in the incidence of HSIL were detected between groups (*p*<0.001, Figure 2). The 5-year cumulative incidence rates of HSIL were 34.0% (95% CI: 17.3–59.8%) among HPV-58 positive cases, 28.0% (95% CI: 13.8–51.6) among HPV-16 positive cases, 5.5% (95% CI: 2.1–14.0%) among women who were positive for the ten other types of HR-HPV, and 0% among women with low/undetermined risk HPV types. Women with HPV-58 demonstrated a higher hazard ratio (hazard ratio = 5.260, 95% CI: 1.538–17.987, *p* = 0.008) for the development of HSIL than women with HPV-16 (hazard ratio = 3.822, 95% CI: 1.176–12.424, *p* = 0.026) in comparison with women with the other ten types of HR-HPV (Table 2). In the multivariate analysis, persistent infection (hazard ratio = 14.248, 95% CI: 1.861–109.115, *p* = 0.011) and HPV-58 (hazard ratio = 4.244, 95% CI: 1.013-17.780, *p* = 0.048) were independently associated with HSIL development in women with HR-HPV infection (Table 2).

## Discussion

The management of women who present with HR-HPV infections and negative cytological results is an important issue. The current recommendation for these cases is HPV and cytology co-testing every 12 months or immediate HPV genotype-specific testing for HPV-16 alone or for HPV-16 and/or -18. The present results suggest that the detection of persistent HPV infection is critical for the prediction of CIN 2/3 in HPV-positive and cytology-negative women. Our results clearly demonstrate that repeated HPV DNA testing for the detection of persistent infection is a practical method that can be used to independently predict HSIL in women with negative cytological results, despite the fact that current HPV DNA tests cannot distinguish between true persistent infection with prevalent HPV and incidental infection by the same HPV type (redetection of the same genotype). Thus persistent infection in this study is equivalent to either uncleared HPV infection or reinfection with the same genotype. Of the 63 women with persistent HR-HPV infection described in the present study, 15 (23.8%) developed HSIL during the follow-up period. However, of 63 HR-HPV positive cases with no persistent infection, only 1 (1.6%) developed HSIL during follow-up. Women with persistent infection but not HSIL may have repeated infections that are not persistent, or HPV is eliminated after ≥ 1 year, or these patients may only be receiving short-term follow-up examinations.

HR-HPV status, especially HPV-16 and/or -18, is an important predictor of HSIL+ in women with negative cytology [13], [14], [15]. The 10-year cumulative incidence rate of HSIL+ in women ≥ 30 years of age with negative cytology was 20.7% (95% CI: 8.6–32.8%) and 17.7% (95% CI: 0.0–36.0%) for HPV-16 positive and HPV-18 positive women, respectively [14]. A study by Kjaer et al. [15] reported that the estimated probabilities of developing HSIL+ in cytology-negative women who are HPV-16 and -18 positive are 26.7% (95% CI: 21.1–31.8%) and 19.1% (95% CI: 10.4–27.3%), respectively, after studying a population for 12 years. In this study, the 5-year cumulative incidence rate of HSIL in women ≥ 30 years of age with negative cytological results who were positive for HPV-16 and/or -18 was 21.2% (95% CI: 10.2–40.9%), but it was 15.8% (95% CI: 8.9–27.2%) among women with other types of HR-HPV. There is a discrepancy between the cumulative incidence rates of HSIL in women with other types of HR-HPV (non-HPV-16 and/or -18) because the 10-year cumulative incidence rate of HSIL+ was only 1.5% (95% CI: 0.3–2.7%) among HR-HPV positive women who were negative for HPV-16 and/or -18 according to the study by Khan et al. [14]. This discrepancy could be explained by the high prevalence of HPV-58 and its significant association with HSIL in this study.

The high prevalence of HPV-58 has been reported in East Asia, including China (28% in Shanghai, 10% in Hong Kong, and 10% in Taiwan), Korea (16%) and Japan (8%) [9], [17], [18]. Although the higher prevalence of HPV-58 in East Asia is not fully understood, genetic characteristics and HPV-58 variants with different oncogenicity are thought to play a role [9], [19]. A relatively high contribution of HPV-58 toward the development of squamous cell carcinoma in East Asia has been reported [17], [18], [20], [21], [22], [23], [24]. Furthermore, HPV-58 has been found in a relatively high proportion of patients with high-grade cervical dysplasia in China [25]. In Korean women, HPV-58 is the second most common type of HPV in women with abnormal cervical cytology [12]. In this study, we show that HPV-58 infection is associated with a high risk of developing HSIL among women with negative cytological results. We also demonstrate that HPV-16 and/or -18 detection alone is insufficient for predicting the development of HSIL in HPV-positive, cytology-negative women and the inclusion of HPV-58 detection in conjunction with HPV-16 and/or -18 is essential for improving the prediction of HSIL in a country with a high prevalence of HPV-58. In this study, all of the cases of HSIL were found within 5 years. This may be partly associated with stopping data collection when women received negative biopsy results because HSIL develops from the normal cervix over a long period of time. However, early presentation of HSIL may be of importance to the development of guidelines recommending immediate HPV genotype-specific testing (for HPV-16 alone or for HPV-16 and/or -18) for women with HR-HPV positive but cytology-negative results [4]. In this study, we emphasize the high oncogenicity of HPV-58 for developing HSIL. Furthermore, these findings are important in the era of HPV vaccination for the prevention of cervical cancer. Our findings also suggest that HPV-58 should be included in HPV vaccination regimens, especially in a high prevalence country.

In conclusion, the present results suggest that the detection of prevalent and persistent HPV infection might be a promising strategy for the management of patients who test positive for HR-HPV but demonstrate negative cytological results. Furthermore, the high association between HPV-58 and developing HSIL indicates that the identification of the HPV-58 genotype is important for the management of women who are HPV-positive and cytology-negative in a country with a high prevalence of HPV-58 infection.
